# Acylated and unacylated ghrelin directly regulate ß-3 stimulated lipid turnover in rodent subcutaneous and visceral adipose tissue ex vivo but not in vivo

**DOI:** 10.1080/21623945.2018.1528811

**Published:** 2018-10-09

**Authors:** Daniel T. Cervone, Justin Sheremeta, Emily N. Kraft, David J. Dyck

**Affiliations:** Department of Human Health and Nutritional Sciences, University of Guelph, Guelph, Ontario, Canada

**Keywords:** lipolysis, reesterification, metabolism, fatty acids, endocrinology, orexigenic, growth hormone, signaling, adrenergic, organ culture

## Abstract

Ghrelin has garnered interest as a gut-derived regulator of lipid metabolism, beyond its classical roles in driving appetite and growth hormone release. Ghrelin’s circulating concentrations follow an ultradian rhythm, peak immediately before a meal and point towards a potential metabolic role in reducing the mobilization of fatty acid stores in preparation for the storage of ingested food. Here, we demonstrate that both acylated and unacylated ghrelin have physiological roles in attenuating lipolysis in mature subcutaneous and visceral adipose tissue depots of rats. Ghrelin blunted the ß3-induction (CL 316, 243) of glycerol release (index of lipolysis) which coincided with a reduced activation of the key lipid hydrolase HSL at two of its serine residues (Ser^563^/^660^). Furthermore, ghrelin appeared to inhibit fatty acid reesterification in the presence of CL such that fatty acid concentrations in the surrounding media were maintained in spite of a reduction in lipolysis. Importantly, these aforementioned effects were not observed following ghrelin injection in vivo, as there was no attenuation of CL-induced glycerol release. This highlights the importance of exercising caution when interpreting the effects of administering ghrelin in vivo, and the necessity for uncovering the elusive mechanisms by which ghrelin regulates lipolysis and fatty acid reesterification. We conclude that both acylated and unacylated ghrelin can exert direct inhibitory effects on lipolysis and fatty acid reesterification in adipose tissue from rats. However, these effects are not observed in vivo and outline the complexity of studying ghrelin’s effects on fatty acid metabolism in the living animal.

## Introduction

Ghrelin is a gut-derived hormone classically defined by its central effect as a potent appetite stimulant.^1,2^ Beyond this, given that its pulsatile secretion coincides with anticipated meal times,^3^ ghrelin has recently harbored interest as a potential regulator of carbohydrate and lipid metabolism. Although convincing evidence exists to suggest that ghrelin administration may influence peripheral tissue metabolism in skeletal muscle and adipose tissue,^4,5^ acute and chronic ghrelin administration are confounded by secondary effects. Acutely, ghrelin can cause growth hormone (GH) and glucagon-like peptide (GLP-1) release, whereas chronic ghrelin administration leads to alterations in body and fat mass due to changes in feeding behavior.^1,6,7^ The assessment of the direct metabolic effects of acylated (AG) and unacylated (UnAG) ghrelin on tissues such as muscle and adipose is relatively sparse.

Adipose tissue (AT) lipolysis releases free fatty acids for utilization by metabolically active tissues, including skeletal muscle, during states of fasting allowing for glucose to be spared for the brain and other glucose-dependent tissues. When a meal is consumed, lipolytic activity in AT is attenuated to prioritize the clearance and storage of plasma fatty acids.^8^ Ghrelin reaches its peak circulating concentration immediately prior to meal ingestion, which points towards a potential role in contributing to the down-regulation of AT lipolysis. Ghrelin may facilitate this transition following a meal, i.e. suppression of lipolysis, when there would no longer be a requirement to preferentially use fatty acids and spare blood glucose. Interestingly, during the prolonged absence of food, the normal rise and fall in ghrelin is largely abolished, at a time when it would be beneficial to sustain elevated AT lipolysis.^3^

Whole-body and isolated tissue models assessing ghrelin’s effects on AT lipolysis have been conflicting. Vestergaard et al. demonstrated that the administration of ghrelin in humans stimulated glycerol release (index of lipolysis) into the interstitial space of subcutaneous AT and skeletal muscle.^5^ However, these findings are potentially confounded by the influence of ghrelin on GH and incretin response. Growth hormone, specifically, has been demonstrated by some to stimulate lipolysis when administered in vivo.^9^ More direct approaches using primary adipocytes have yielded results that contrast with the aforementioned findings, and demonstrate an inhibitory role for ghrelin on the β-adrenergic stimulation of lipolysis.^10^ To date, the direct effects of ghrelin have not been assessed in isolated mature AT, difference in subcutaneous and visceral AT responses have not been examined, and there has been very little determination of UnAG’s role in AT lipolysis, particularly in vivo. This is important, given the growing evidence suggesting that UnAG may also have metabolic effects.^11^ Moreover, the acute measurement of lipolysis following ghrelin administration in rats is lacking, and the underlying cellular signaling events to clarify the regulatory role of both ghrelin isoforms on lipolysis remain to be investigated.

Therefore, in the current study, we aimed at extending previous findings to elucidate whether AG and UnAG could regulate lipolysis directly (ex vivo) in mature subcutaneous and visceral adipose tissue depots from rodents, while also assessing concomitant changes in cellular signaling. In addition, we examined whether any of the observed lipolytic or signaling effects observed ex vivo would also be evident following acute in vivo ghrelin administration with the β3-receptor specific pharmacological agonist CL 316,243. The β3-receptor is highly expressed in adipose tissue, but not skeletal muscle or liver.^12^

## Methods

### Animals

All procedures were approved by the Animal Care Committee at the University of Guelph and followed Canadian Council of Animal Care guidelines. Male Sprague-Dawley rats were obtained from Charles River laboratories (Québec, ON, Canada) at approximately 4–5 weeks of age (~ 200-250g). Rats were given ad libitum access to regular chow food and water and allowed to acclimatize upon arrival for one week. To avoid high endogenous levels of circulating ghrelin as a potentially confounding factor, food was reintroduced (for less than 1h) prior to experiments following an overnight fast, and it was visually confirmed that the food was consumed. Rodents were anesthetized with an intraperitoneal injection of sodium pentobarbital (6mg per 100g body mass) prior to all surgical procedures. Tissues were excised for incubation, and in vivo administration of ghrelin occurred 2-3h following the removal of food.

### Materials and reagents

Reagents, molecular weight markers and nitrocellulose membranes were purchased from BioRad (Mississauga, ON, Canada). Western lighting plus enhanced chemiluminescence (ECL) was purchased from Perkin-Elmer (NEL105001EA). The following antibodies were purchased from Cell Signaling: total-AMPK (Cat. No. 2532), phospho-AMPK (Cat. No. 2535), total-ACC (Cat. No. 3676), phospho-ACC (Cat. No. 11818), total-HSL (Cat. No. 4107), total-ERK (Cat. No. 4695), phospho-ERK (Cat. No. 4370), phospho-HSL 660, 565, 563 (Cat. Nos. 4126, 4137, 4139). The following antibodies were purchased from Abcam: total-ATGL (Cat. No. 109251), phospho-ATGL (Cat. No. 135093). NP40 cell lysis buffer (Cat. No. FNN0021) was obtained from Life Technologies and PMSF and protease inhibitor were obtained from Sigma (Cat. Nos. 78830 and 9599). Insulin (Humulin rDNA origin) was purchased from Eli Lilly (Toronto, ON, Canada). Recombinant human growth hormone protein was purchased from Abcam (Cat. No. ab116162). Acylated (Cat. No. H-4862) and unacylated (Cat. No. H-6264) ghrelin were sourced from Bachem (Torrance, CA, USA). For adipose tissue incubations, fatty-acid free bovine serum albumin (Cat. No. 10775835001), Medium 199 (Cat. No. M7653), L-glutamine (Cat. No. G7513), dexamethasone 21-phosphate disodium salt (Cat. No. D1159) and CL 316,243 (Cat. No. C5976) were all sourced from Sigma. Antibiotic/antimycotic solution (Cat. No. 30004Cl) was purchased from Corning.

### Ex vivo (Adipose Tissue Organ Culture – ATOC) lipolysis and signaling

Following anesthesia, surgeries were carefully performed to excise both subcutaneous (inguinal) and visceral (retroperitoneal) adipose tissue which was immediately placed into 5mM glucose M199 media supplemented with 1% antibiotic/antimycotic, 50 μU insulin and 2.5 nM dexamethasone. 250mg portions were snipped, minced in petri dishes and covered with 3.5mL of ATOC media after which they remained incubated under warmed (37°C), humidified and gassed (95% O2, 5% CO2) conditions for 24h. Following 24h pre-treatment, media was swapped with fresh media to which respective treatments (sterile H_2_O, 150ng/ml AG, 150ng/ml UnAG, 250ng/mL GH, or 1µM CL) were added. Previous work has found that these doses for AG, UnAG and CL elicit significant metabolic effects; the dose used for GH represents a concentration that is achieved in response to ghrelin administration in rats.^7,10,13^ Sampling of media for the quantification of glycerol and free fatty acids was done at t = 2, 4 and 8h. Aliquots were immediately frozen at −80°C for further analysis. CL significantly increased glycerol accumulation (subcutaneous depot shown, for simplicity) compared to control at each time point (t = 2h: Con, 1.0 ± 0.1 vs. CL, 2.3 ± 0.2; t = 4h: Con, 1.0 ± 0.3 vs. CL: 3.3 ± 1.0; t = 8h: Con, 1.0 ± 0.2 vs. CL, 2.9 ± 0.8 mM/g). Given that the accumulation was significant at 2h and was not linear beyond this point, 2h was chosen as the time point of collection for all future measurements of lipolysis. For the purpose of assessing signaling proteins in adipose tissue samples from ATOC, tissue was rinsed in ice cold phosphate-buffered saline (PBS) following 2h of incubation with treatment, snap frozen in liquid nitrogen, and stored at −80°C until further processing for western blotting.

### In vivo lipolysis and signaling

Following anesthesia, rats were administered an intraperitoneal injection of either CL, CL+ AG or CL+ UnAG, or a body mass-adjusted bolus of saline (all 1mg/kg). Other in vivo work^4^ has demonstrated significant metabolic and physiological effects using a similar injection dose of ghrelin. Also, previous work in our lab^13^ has demonstrated a robust increase in lipolysis following CL administration by 30 min. As such, this time point was used for blood and tissue collection. Specifically, a cardiac puncture was performed to obtain terminal blood samples. Blood rested on ice for an additional 30 min and was then centrifuged for 10 min at 1500g. Serum was collected and immediately snap frozen in liquid nitrogen and stored at −80°C for further processing. For in vivo signaling, subcutaneous (inguinal) and visceral (retroperitoneal) adipose tissue were excised 30min post-injection, rinsed in saline, blotted dry and frozen in liquid nitrogen to be stored at −80°C.

### Glycerol quantification

Glycerol was quantified from ATOC media and serum samples via single-wavelength spectrophotometry using commercially available reagents from Sigma derived from their glycerol assay kit. These reagents included glycerol standard (Cat. No. G7793) and glycerol reagent (Cat. No. F6428).

### Non-esterified fatty acid (NEFA) quantification

NEFAs were quantified from ATOC media and serum samples using commercially available reagents (Cat. Nos. 995-34791, 993-35191, 999-34691, 991-34891) and standards (Cat. No. 276-76491) from Wako Diagonistics (Richmond, VA), which are included in their free fatty acid assay kit.

### Primary fatty acid reesterification

Primary fatty acid reesterification was calculated as the difference between the theoretical fatty acid release (3 x glycerol release into media/serum) and the measured fatty acid release. This assumes negligible rates of fatty acid oxidation in adipose tissue and describes a crude measure for the absolute rates of fatty acid reesterification, as described previously by Van Harmelen et al.^14^

### Circulating insulin quantification

Insulin in serum was determined using a commercially available ELISA kit from Millipore Sigma (Cat. No. EZRMI-13K) as described previously.^15^ Accuracy was validated using 2 quality control standards provided with the kit.

### Western blotting

Adipose tissue (~ 250-300mg) was placed directly into homogenization tubes containing lysis beads prior to being frozen in liquid nitrogen. Protease inhibitor and PMSF were then added, after which samples were homogenized and centrifuged at 4°C. The supernatant was then carefully removed (to not disrupt the fat layer) and transferred to a new tube. BCA assays were performed to determine protein content from homogenized samples to facilitate subsequent sample preparation for western blots.^16^

For western blots, equal amounts of sample protein (10μg) were loaded onto 10% gels (5% for ACC), as we have published previously.^17^ Samples were then transferred for 1h at 100V onto nitrocellulose membranes and blocked in non-fat skim milk powder and TBST. Membranes were then incubated at 4°C overnight in primary antibody supplemented with 5% BSA/5% skim milk powder as per manufacturer guidelines. Following primary incubation, membranes were washed with TBST and exposed to secondary (anti-rabbit) antibody (1:2000) for 1h at room temperature and then washed again (2x TBST, 1x TBS). Protein bands were visualized using ECL and quantified using densitometry on Alpha Innovate Software. All membranes were ponceau stained and quantified to assess equal loading and blots were normalized to their respective ponceau image. Western blots are presented as arbitrary units of normalized phosphorylated/total protein.

### Statistics

All data are expressed as mean ± standard error. A repeated measure (by both factors – with or without CL or ghrelin) 2-way analysis of variance (ANOVA) was performed for ex vivo ATOC experiments. Pending significance with the ANOVA, a Tukey’s multiple comparisons post-hoc test was used to find any main (CL or AG/UnAG) or interaction (CL x AG/UnAG) effects of treatments. A repeated measure, non-parametric one-way ANOVA (Friedman’s test) was performed for the GH-treated subsets of ex vivo ATOC experiments as well as all in vivo injection and western blotting experiments. If significance was detected with the ANOVA, multiple comparisons were assessed using a Dunn’s post-hoc test. In all figures, letters were used to denote statistical significance, such that groups sharing a common letter are not significantly different from each other. Data was considered significant at p < 0.05.

## Results

### Ex vivo, ATOC experiments

#### Ex vivo, AG and UnAG do not independently affect AT lipolysis or fatty acid reesterification

**Figure 1.**
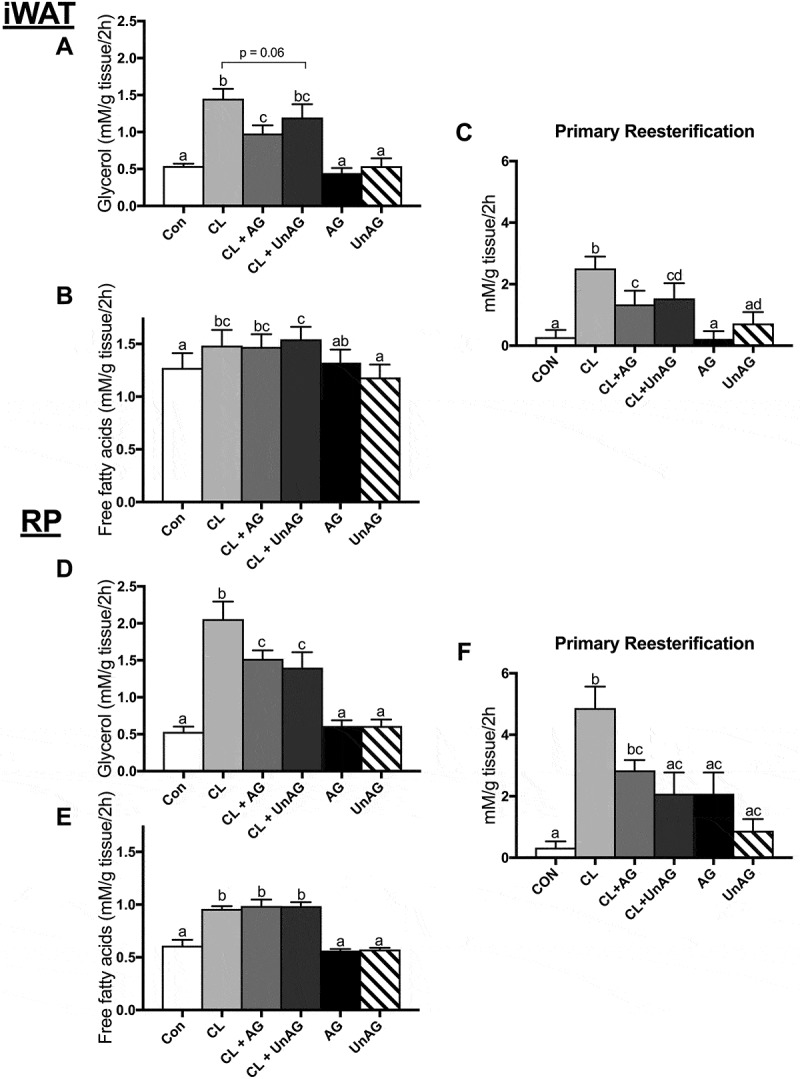
The *ex vivo* effects of CL and CL + ghrelin combination treatments on lipolysis as measured by glycerol (a and d) and free fatty acid (b and e) release and fatty acid reesterification (c and f) in subcutaneous inguinal white (top) and visceral retroperitoneal (bottom) adipose tissue. Data were analyzed using a repeated measures two-way ANOVA (n = 9–12) and expressed as mean ± standard error, in mM/g tissue/2h. Data sharing a letter are not statistically different from each other. p < 0.05 was considered statistically significant.

During ATOC incubations with iWAT and RP, neither AG nor UnAG had a significant effect on indices of lipolysis i.e. glycerol (Figure 1(a,d)) and free fatty acid release (Figure 1(b,f)). Calculated fatty acid reesterification (Figure 1(c,f)) was also unaffected. Given the lack of any independent ghrelin effect on these functional measurements, AG and UnAG treatments were not further pursued for signaling purposes.

#### Ex vivo, CL-stimulated glycerol release and fatty acid reesterification are attenuated by AG and UnAG

CL treatment independently stimulated lipolysis (Figure 1(a-e)). This was evidenced in both fat depots by an increase in glycerol (iWAT, ~ 2.7-fold, p < 0.01; RP, ~ 3.9-fold, p < 0.01) and free fatty acid (iWAT, ~ 1.2-fold, p < 0.05; RP, ~ 1.6-fold, p < 0.01) release. CL’s stimulatory effect on glycerol release was significantly attenuated (p < 0.05) by AG in both (iWAT: Figure 1(a) and RP: Figure 1(d)) AT depots, whereas UnAG had a significant effect in RP (Figure 1(d)) and trended (p = 0.06) towards a reduction in iWAT (Figure 1(a)). Interestingly, AG and UnAG did not suppress CL’s ability to increase fatty acid release from either fat depot (Figure 1(b)) and RP (Figure 1(e)). A large proportion of hydrolyzed fatty acids are reesterified back into TAGs; accordingly, given CL’s stimulatory effect on lipolysis, CL also significantly increased fatty acid reesterification in both depots (Figure 1(c,f)). AG and UnAG significantly (p < 0.01) blunted this reesterification response to CL in iWAT (Figure 1(c)), whereas only CL+ UnAG was significant in RP (Figure 1(f)).

#### Ex vivo, GH alone or in combination with AG and UnAG, does not affect AT lipolysis

**Figure 2.**
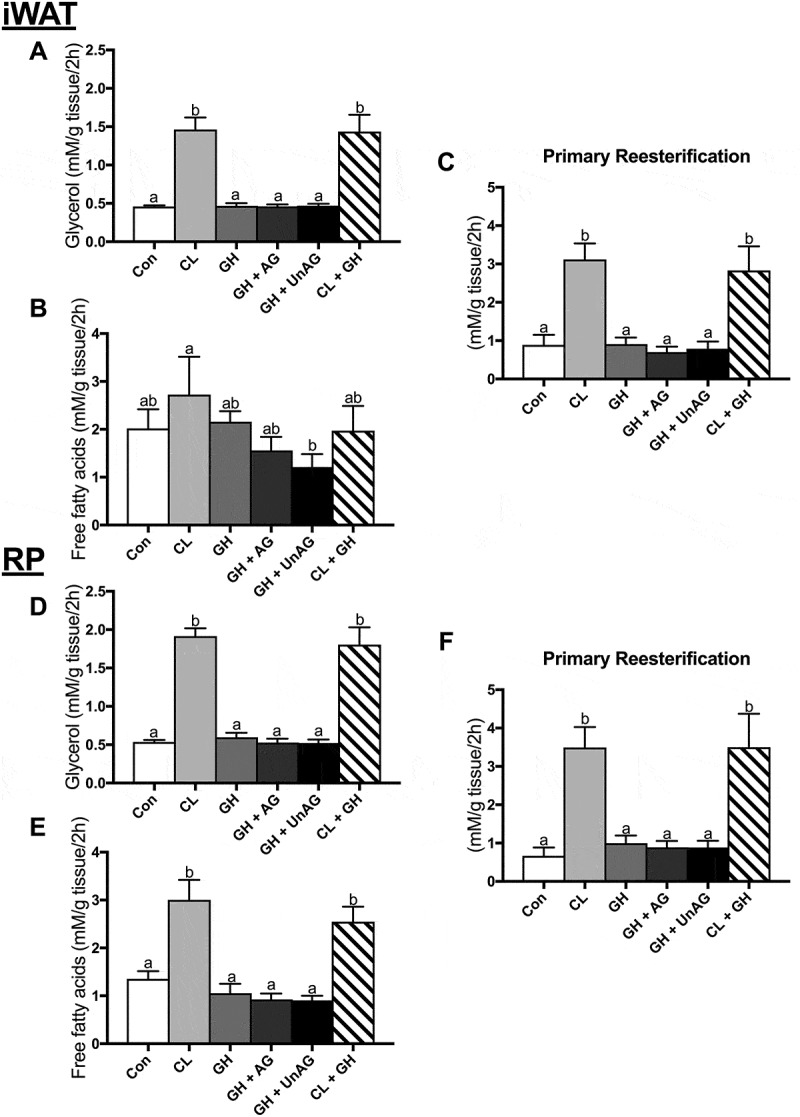
The *ex vivo* effects of GH and GH + ghrelin or CL combination treatments on lipolysis as measured by glycerol (a and d) and free fatty acid (b and e) release and fatty acid reesterification (c and f) in subcutaneous inguinal white (top) and visceral retroperitoneal (bottom) adipose tissue. Data were analyzed using a repeated measures one-way ANOVA (n = 6–8) and expressed as mean ± standard error, in mM/g tissue/2h. Data sharing a letter are not statistically different from each other. p < 0.05 was considered statistically significant.

Given AG’s physiological role in stimulating GH release, we determined whether ghrelin could modulate GH’s purported role as a stimulator of AT lipolysis.^9,18^ However, with our acute (2h) treatment protocol, when compared to control, GH did not alter glycerol (Figure 2(a,d)) or free fatty acid (Figure 2(b,e)) release alone or in combination with CL in iWAT or RP. CL significantly increased (p < 0.001) glycerol release in iWAT (Figure 2(a)) and RP (Figure 2(d)) as expected. CL also significantly increased (p < 0.001) free fatty acid release in RP (Figure 2(e)) but not iWAT within this subset of incubations (Figure 2(b)). Furthermore, AG and UnAG did not elicit any effects when combined with GH, as lipolytic endpoints were not significantly different from control or GH alone (Figure 2(a-e)). Only the CL-treated AT, which showed an increase in lipolysis, demonstrated an increase in fatty acid reesterification (Figure 2(c,f)). Given the lack of any direct, acute effect on the functional measurements of lipolysis and fatty acid reesterification, further experiments with GH were not pursued for determination of protein signaling.

#### Ex vivo, CL-stimulated HSL phosphorylation is attenuated with both AG and UnAG

**Figure 3.**
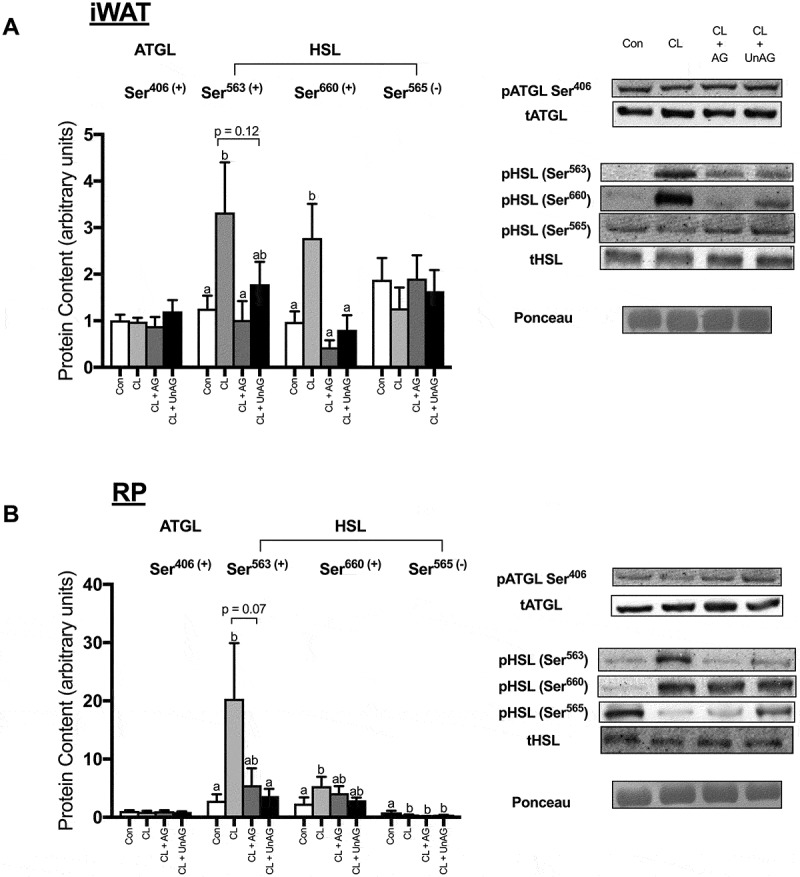
The *ex vivo* effects of CL and CL + ghrelin combination treatments on the activation of lipolytic enzymes ATGL and HSL in subcutaneous inguinal white (a) and visceral retroperitoneal (b) adipose tissue. Data were analyzed using a repeated measures one-way ANOVA (n = 6–12) and expressed as mean ± standard error, in arbitrary protein units (phospho/total). Data sharing a letter are not statistically different from each other. p < 0.05 was considered statistically significant.

CL significantly increased the phosphorylation of HSL at activating Ser^563/660^ residues in both iWAT and RP (Figure 3(a,b); p < 0.01). CL treatment also significantly reduced the phosphorylation of HSL at its inhibitory Ser^565^ residue, but this effect was only observed in RP (Figure 3(b)). In iWAT, AG blunted the CL-mediated increase in HSL phosphorylation at both Ser^563^ and Ser^660^ (p < 0.05), whereas UnAG only reduced HSL activation at Ser^660^ (Figure 3(a)). In RP, both AG (p = 0.07) and UnAG (p < 0.05) attenuated CL’s activation of HSL at Ser^563^ but had no significant effect at Ser^660^ (Figure 3(b)). In the presence of CL, AG and UnAG did not further alter HSL phosphorylation at its inhibitory Ser^565^ site in either adipose tissue depot (Figure 3(a,b)). The activation of ATGL (a TAG hydrolase) at Ser^406^, was unaltered by all treatments in both iWAT (Figure 3(a)) and RP (Figure 3(b)).

#### Ex vivo, AMPK, ACC and ERK activation remained unaltered by CL and ghrelin treatments

**Figure 4.**
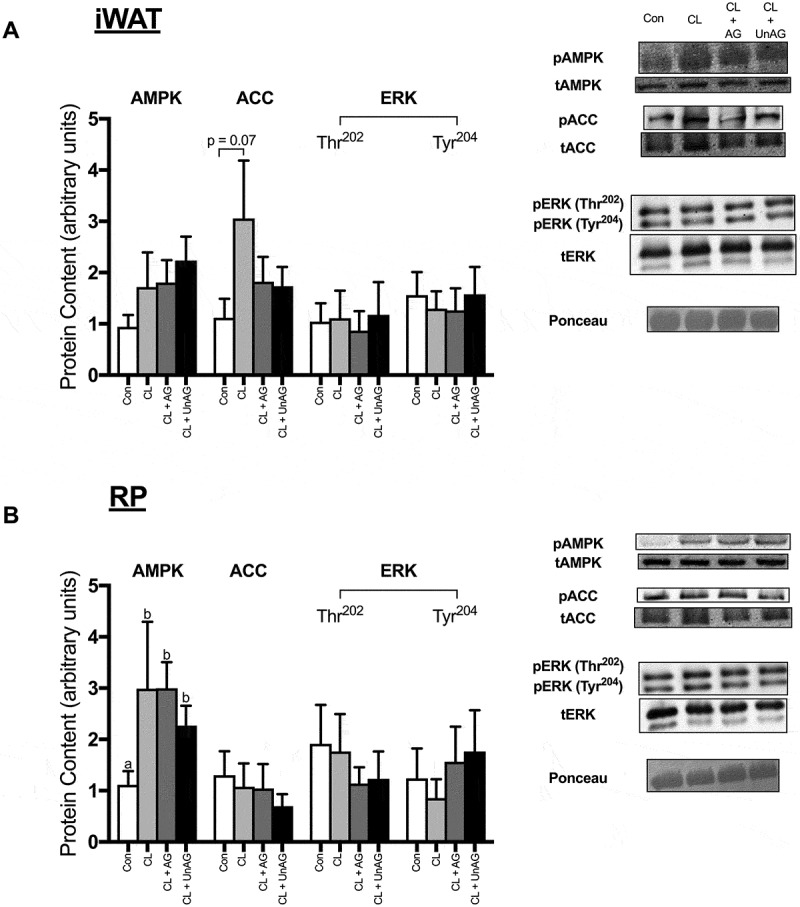
The *ex vivo* effects of CL and CL + ghrelin combination treatments on the activation of ERK, the cellular energy-sensing enzyme AMPK and its downstream target ACC in subcutaneous inguinal white (a) and visceral retroperitoneal (b) adipose tissue. Data were analyzed using a repeated measures one-way ANOVA (n = 8–10) and expressed as mean ± standard error, in arbitrary protein units (phospho/total). Data sharing a letter are not statistically different from each other. p < 0.05 was considered statistically significant.

AMPK Thr^172^ phosphorylation was significantly (p < 0.05) elevated following all CL treatments, but only in RP (Figure 4(b)), but this did not translate to its downstream target ACC, as its Ser^79^ phosphorylation was unaffected. No consistent treatment effects on AMPK or ACC activation were apparent in iWAT (Figure 4(a)). ERK phosphorylation at its Thr^202^ and Tyr^204^ residues were not significantly different between any groups in iWAT or RP (Figure 4(a,b)).

### In vivo injection experiments

#### Stimulation of lipolysis with CL is not blunted with AG and UnAG in vivo

**Figure 5.**
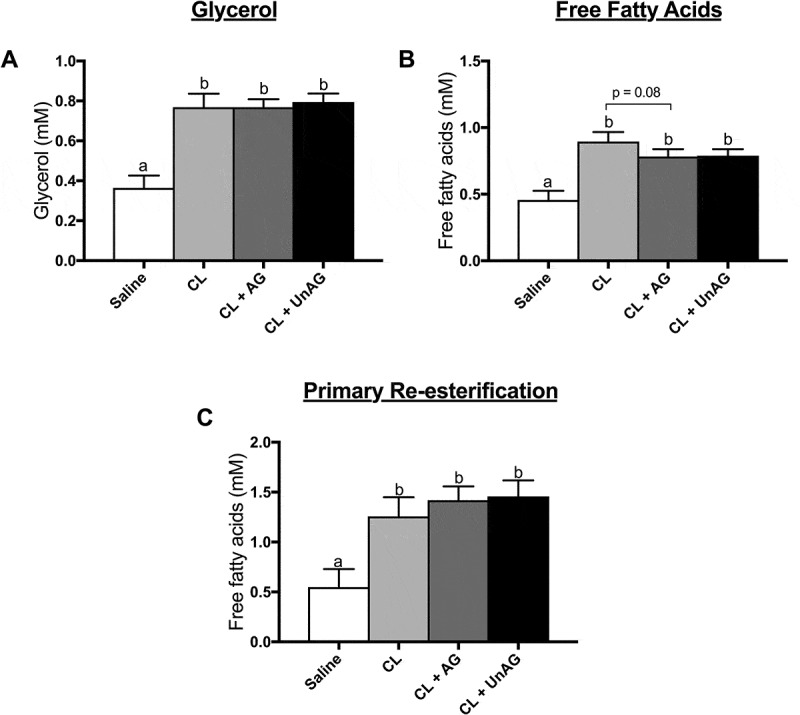
The *in vivo* effects of CL and CL + ghrelin co-injections on lipolysis as measured by circulating glycerol (a) and free fatty acid (b) release and fatty acid reesterification (c). Data were analyzed using a repeated measures one-way ANOVA (n = 7–8) and expressed as mean ± standard error, in mM. Data sharing a letter are not statistically different from each other. p < 0.05 was considered statistically significant.

CL-injection significantly (p < 0.01) increased lipolysis in vivo, as shown by a 2-fold rise in both whole-body circulating glycerol (Figure 5(a)) and free fatty acids (Figure 5(b)), compared to saline control. Similarly, all in vivo CL-injections showed a significant (p < 0.05) increase in fatty acid reesterification (Figure 5(c)). Contrary to our direct, ex vivo (ATOC) findings, co-injection of AG or UnAG with CL did not alter circulating glycerol (Figure 5(a)) or fatty acid (Figure 5(b)) concentrations when compared to CL alone. AG trended (p = 0.08) towards reducing CL-stimulated free fatty acid release (Figure 5(b)).

#### In vivo, neither AG nor UnAG blunt CL’s activation of HSL

**Figure 6.**
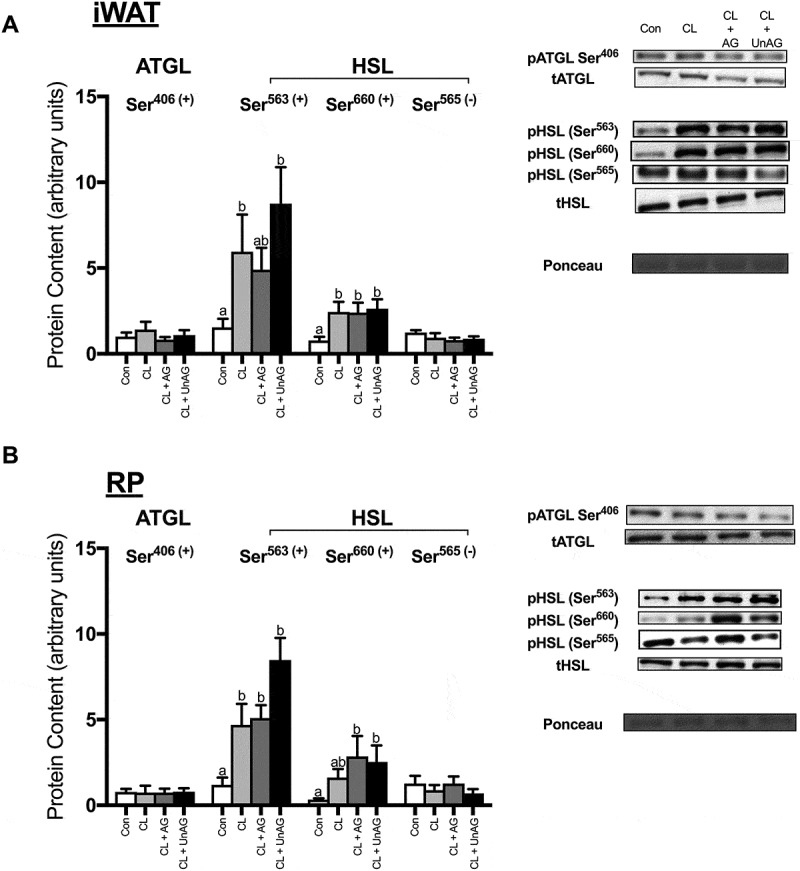
The *in vivo* effects of CL and CL + ghrelin co-injections on the activation of lipolytic enzymes ATGL and HSL in subcutaneous inguinal white (a) and visceral retroperitoneal (b) adipose tissue. Data were analyzed using a repeated measures one-way ANOVA (n = 6–8) and expressed as mean ± standard error, in arbitrary protein units (phospho/total). Data sharing a letter are not statistically different from each other. p < 0.05 was considered statistically significant.

CL-injection significantly increased the phosphorylation of HSL at both Ser^563^ and Ser^660^ residues in iWAT (Figure 6(a); p < 0.05); in RP, only Ser^563^ phosphorylation was increased by in vivo CL-injection. The phosphorylation of HSL at activating Ser^563/660^ residues following co-administration of CL with AG and UnAG were not significantly reduced from CL alone in either AT depot (Figure 6(a,b)). The phosphorylation of HSL at its inhibitory Ser^565^ residue was unchanged in both iWAT (Figure 6(a)) and RP (Figure 6(b)) following all injections. The activation of ATGL was unaffected by all treatments (Figure 6(a,b)).

#### In vivo, AMPK and ACC activation are unaltered by CL and ghrelin

**Figure 7.**
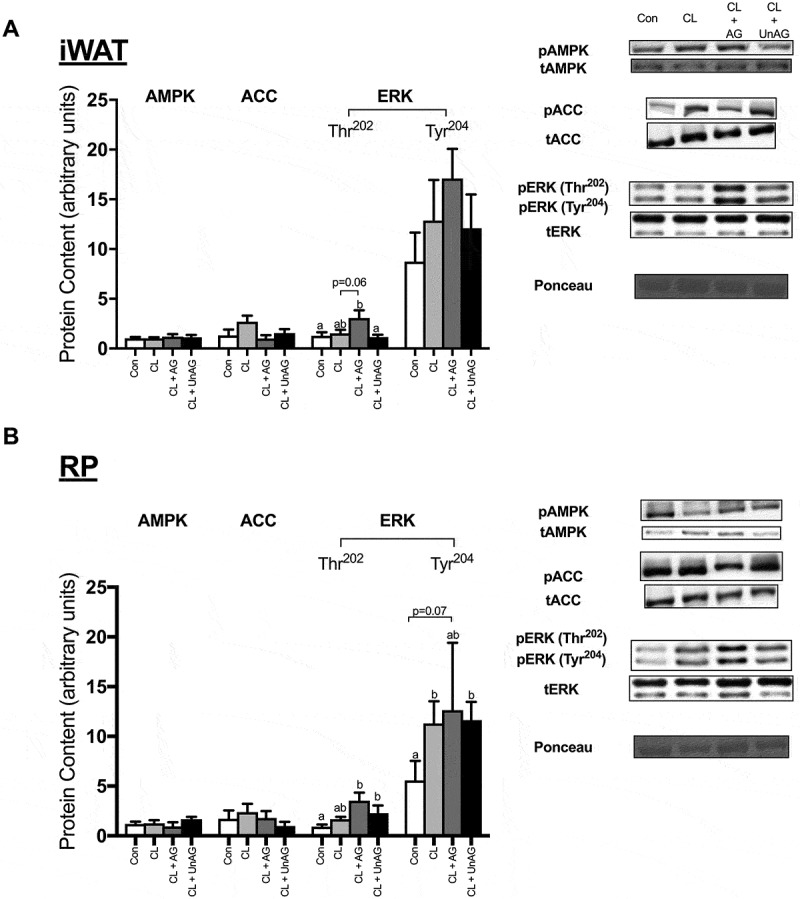
The *in vivo* effects of CL and CL + ghrelin co-injections on the activation of ERK, the cellular energy-sensing enzyme AMPK and its downstream target ACC in subcutaneous inguinal white (a) and visceral retroperitoneal (b) adipose tissue. Data were analyzed using a repeated measures one-way ANOVA (n = 6–8) and expressed as mean ± standard error, in arbitrary protein units (phospho/total). Data sharing a letter are not statistically different from each other. p < 0.05 was considered statistically significant.

AMPK (Thr^172^) and its downstream target ACC (Ser^79^) were not statistically different between any injection treatment in either adipose tissue depots (Figure 7(b,c)).

#### In vivo, ERK phosphorylation is variably increased with CL and ghrelin

Injection of saline, CL and CL+ UnAG had no effect on the activation of ERK in iWAT (Figure 7(a)). Interestingly, co-administration of CL with AG significantly (p < 0.05) increased the activation of ERK at its Thr^202^ but not Tyr^204^ residue in iWAT (Figure 7(a)). All CL-stimulated treatments had a stimulatory effect on ERK activation at its Tyr^204^ residue in RP (Figure 7(b)). AG and UnAG further increased ERK phosphorylation at Thr^202^ beyond CL alone (Figure 7(b)).

#### In vivo injections of CL increase insulin, but do not affect AT AKT phosphorylation

CL injection has been reported to cause insulin secretion in vivo;^19^ therefore we measured insulin following injections. All CL-injections significantly increased circulating insulin concentrations compared to saline (Table 1). Interestingly, the co-injection of UnAG with CL yielded a significantly greater rise in insulin when compared to CL alone. However, these concentrations were not sufficient to blunt CL’s effects in stimulating lipolysis. This is likely due to the fact that AKT phosphorylation, a mediator of insulin action in peripheral tissues and inhibitor of lipolysis, was unaltered. Despite the CL-mediated increases in serum insulin, the phosphorylation of AKT at its Ser^473^ and Thr^308^ residues was unaffected by treatments in iWAT and RP (Table 1).

**Table 1.** The in vivo effect of CL and CL + ghrelin combination treatments on AKT phosphorylation (a marker of insulin signaling activation) in subcutaneous iWAT and visceral RP adipose tissue depots (arbitrary units, phospho/total, normalized for ponceau) and serum insulin concentrations (ng/ml). Data are expressed as mean ± standard error. Data were analyzed using a repeated measures one-way ANOVA (n = 6–8). Data sharing a letter are not statistically different from each other. p < 0.05 was considered statistically significant.

	iWAT	RP
pAKT (Ser473)		
pAKT (Thr308)		
Total AKT		
Ponceau		

*Adipose Tissue pAKT*		Saline (control)	CL 316,243	CL + AG	CL + UnAG
iWAT	**Ser^473^**	1.1 ± 0.47	0.84 ± 0.26	1.52 ± 0.34	1.48 ± 0.43
	**Thr^308^**	0.99 ± 0.33	1.89 ± 0.94	1.59 ± 0.53	2.69 ± 1.58
RP	**Ser^473^**	0.94 ± 0.17	1.51 ± 0.45	1.55 ± 0.21	2.15 ± 0.59
	**Thr^308^**	1.59 ± 0.64	1.18 ± 0.30	3.01 ± 1.32	2.38 ± 1.11
*Serum Insulin*		4.6 ± 0.6^a^	8.1 ± 0.6^b^	9.2 ± 0.7^bc^	11.3 ± 0.5^c^

## Discussion

### Overall finding

Ghrelin spikes immediately prior to entrained mealtimes. We hypothesized that ghrelin would exert an antilipolytic effect which blunt the mobilization of fatty acids in advance of the upcoming meal, thereby facilitating the storage of lipid following meal ingestion. Interestingly, ghrelin’s normal entrained rise and fall are lost during times of prolonged energy deficit,^3^ which would presumably facilitate lipolysis to spare blood glucose. However, a direct role for ghrelin in the regulation of AT lipolysis has not been thoroughly examined. In the current study we demonstrate that both forms of ghrelin may be important regulators of fatty acid turnover in mature rodent adipose tissue (AT). Ex vivo, high doses of AG and UnAG directly attenuated the ability of the ß3-agonist CL 316, 243 to release glycerol from subcutaneous and visceral AT depots. For the first time, lipolytic cellular signaling was extensively explored, which demonstrated that in the presence of CL, both AG and UnAG likely exerted their antilipolytic effects by reducing the phosphorylation of HSL at two of its stimulatory (Ser^563^ and Ser^660^) residues. Interestingly, neither AG nor UnAG reduced media FFA concentration with CL treatment, suggesting that ghrelin may also directly hinder the ability for AT to reesterify fatty acids. To our knowledge, we are the first to demonstrate this. Finally, in vivo, we did not demonstrate any blunting effect of CL-induced increases in circulating FFA or glycerol concentrations subsequent to ghrelin injection. This may be due to confounding secondary factors, although our incubation experiments would suggest that this is not due to the acute action of GH, despite purported as being lipolytic. Our data supports a potential role for ghrelin as a regulator of lipolysis, at least in isolated AT. Further work needs to examine whether this effect is exhibited in vivo, and if not, the mechanisms underlying its disappearance.

### Ghrelin directly inhibits ß3-stimulation of lipolysis, but does not reduce fatty acid concentration in the media

Our data indicate that ghrelin inhibits lipolysis, as evidenced by reduced glycerol appearance in both subcutaneous and visceral AT depots. Importantly, however, ghrelin does not serve this role independently, but only in the presence of adrenergic stimulation (ß-agonist, CL). These are important distinctions that have not been previously assessed.^10,20^ This would be physiologically relevant as ghrelin’s peak concentration would coincide with that of catecholamines stimulating AT mobilization during times of energy deficit. CL-stimulated glycerol release in the presence of ghrelin was still higher than control, suggesting that ghrelin may not be sufficient to completely abolish lipolysis in the absence of other potent antilipolytic stimuli that would increase with the consumption of a meal, such as insulin.^21^ This should be further examined. Ghrelin’s direct effects on fatty acid transport and storage in AT also remain to be studied.

When TAG is completely hydrolyzed, three FFA are released for each glycerol, and either delivered to other tissues via circulation, or reesterified. In our study, unlike glycerol, the increase in media FFA induced by CL was not attenuated by ghrelin. This suggests that ghrelin also inhibited the reesterification of liberated fatty acids, allowing their concentration to remain elevated. While it is logical to speculate that ghrelin may reduce glucose-sparing AT lipolysis prior to mealtime (and the ingestion of glucose), the potential inhibition of reesterification is perplexing. It could be that secondary to its role in reducing AT lipolysis, ghrelin maintains elevated fatty acids via a reduction in reesterification to protect blood glucose in the event that a meal is missed (i.e. glucose is not consumed). Then, upon meal consumption, other hormones (e.g. insulin) can govern lipid turnover in AT. Nevertheless, perhaps at its simplest, the reduction in reesterification simply reflects the observed absolute reduction in lipolysis, given that upwards of ~ 30% of hydrolyzed fatty acids can be cycled back to storage in rats.^22^ It should be noted that we did not directly assess fatty acid reesterification, and future work should employ a more accurate assessment of reesterification with radiolabeled fatty acids, and any underlying mechanisms by which ghrelin may regulate this process.

### Ghrelin alters lipolytic signaling through HSL

Despite some purported effects for ghrelin’s positive^5^ and negative^20^ regulation of AT lipolysis, the ability for ghrelin to directly regulate lipolytic enzyme activity has been sparsely examined. One previous report assessing ghrelin action demonstrated a general trend towards antilipolytic signaling at phosphodiesterase, upstream of HSL.^20^ To our knowledge, the only study examining HSL did not assess its state of phosphorylation, nor did it have any positive control for lipolysis.^23^ Overall, we did not observe independent ghrelin effects on lipolysis. In line with this, HSL phosphorylation was unaffected by independent AG and UnAG treatments (data not shown). Expectedly, HSL activation was significantly decreased with ghrelin’s addition to CL treatment, coinciding with the observed reduction in glycerol release, ex vivo. Although the mechanism mediating the ghrelin-driven reduction in HSL phosphorylation has yet to be tested directly, HSL activity (and subsequent lipolysis) appears to have been reduced by AG and UnAG via its stimulatory (Ser^563^ and Ser^660^), but not inhibitory (Ser^565^) residues.

### Ghrelin does not affect the AMPK/ACC and ERK axes

Until now, there has been no effort towards elucidating potential ghrelin action on other enzymes and pathways thought to contribute to lipolysis (e.g. ATGL, ERK). In the current investigation there were no observed changes in ATGL phosphorylation with any treatment in vivo or ex vivo, although it has been speculated that this covalent modification may not entirely reflect ATGL activity within adipose tissue and that PKA is not likely to be the enzyme responsible for its alteration.^24^ Some evidence would suggest an AMPK requirement for ATGL phosphorylation.^25^ In line with this, we did not observe any alterations in AMPK activation. Extracellular signal-regulated kinases (ERKs), have been previously shown to respond to ß-adrenergic agonists in adipocytes^26,27^ and stimulate lipolysis and HSL activity.^28^ In ATOC experiments, we did not observe changes in ERK activation with CL or ghrelin treatments, although this could be due to the timing of our measurements. Tissue was frozen for assessment of signaling to match out determination of lipolysis, which occurred at 2h and may have missed acute changes in ERK, upstream of HSL modification. In vivo, ERK was activated in visceral, but not subcutaneous AT, following all CL treatments. It is likely that visceral AT responded to a greater degree to the adrenergic stimulus due to its increased ß3-receptor function.^29–31^ Interestingly, the CL+ AG combined injection consistently demonstrated the highest increase in ERK activation, and this treatment significantly activated ERK in iWAT. This could potentially be due to a secondary rise in GH, which has been shown to signal through the ERK pathway in AT.^32^ Clearly, however, this was not sufficient to drive a further increase in lipolysis by 30min, in vivo.

### Ghrelin’s in vivo effects on lipolysis

To our knowledge, we are the first to assess the acute action of ghrelin on lipolysis in rats, in vivo. In the presence of the ß3-agonist CL, ghrelin’s ability to down-regulate AT lipolysis was not observed in vivo. The assessment of the metabolic effects of ghrelin administered in vivo is abundant in literature but has limitations. Acutely, AG has been shown to drive GH release in both rodents^7^ and humans.^33^ This is potentially confounding given that GH, although subject to large interindividual variability, typically elicits an increase in lipolysis in humans,^34^ as well as in cell culture.^18^ As such, we initially sought to determine whether ghrelin could modify the lipolytic response of our ATOC preparation to GH. Unexpectedly, we did not observe any increases in lipolysis with GH. There are other reports on the lack of GH effect on lipolysis in explant^35^ and adipocyte^36^ models. While the mechanistic nature and time-course for GH’s ability to alter lipolysis is beyond the scope of our investigation, it is worthy of further consideration as some have pursued.^36^ Also, it is interesting that others whom have made efforts to control for GH (in vivo) through the use of somatostatin, still observed increases in lipolysis with AG.^5,37^ It must be given deliberation that studies demonstrating a ghrelin-mediated increase in lipolysis in vivo have typically been compared to baseline, or in some cases an antilipolytic hyperinsulinemic clamp as a control.^5^ CL is very potent, and it may be difficult to observe changes in lipolytic endpoints in the context of this pharmacological agent, which could explain why AG did not elicit any increase in lipolysis in vivo in the current study. We also attempted to isolate whole-body lipolytic responses to AT with CL; however, the potential contribution of ghrelin’s effects on lipid metabolism in other tissues cannot be discounted. Also, fatty acid-induced insulin release is a known confounding effect of CL administration,^19^ and could ultimately down-regulate lipolysis. However, we feel that this is unlikely to be a factor in the present study as we found no change in AKT phosphorylation (a marker of insulin signaling) in AT across treatments, and most importantly, CL maintained its ability to significantly stimulate lipolysis.

In conclusion, our data would suggest that both forms of ghrelin can directly reduce ß3-driven lipolysis in mature inguinal white and retroperitoneal adipose tissue depots through a parallel reduction in HSL activation. The cellular nature of UnAG action on peripheral tissues remains elusive, particularly with numerous others suggesting that it may act independently of the GHS-R1a.^38,39^ Nevertheless, these antilipolytic effects will require further testing to uncover any physiological relevance as well as extensive in vivo work to contextualize ghrelin’s role as a regulator of fatty acid metabolism surrounding mealtime. Finally, mechanisms relating to ghrelin’s ability to concurrently inhibit AT fatty acid reesterification should be pursued.
